# Sensory Characteristics and Volatile Components of Dry Dog Foods Manufactured with Sorghum Fractions

**DOI:** 10.3390/molecules22061012

**Published:** 2017-06-17

**Authors:** Brizio Di Donfrancesco, Kadri Koppel

**Affiliations:** Center for Sensory Analysis and Consumer Behavior, Kansas State University, 1310 Research Park Dr, Manhattan, KS 66502, USA; didonfrancescob@ymail.com

**Keywords:** dog food, sensory, sorghum, SPME, volatiles

## Abstract

Descriptive sensory analysis and gas chromatography-mass spectrometry (GC-MS) with a modified headspace solid-phase microextraction (SPME) method was performed on three extruded dry dog food diets manufactured with different fractions of red sorghum and a control diet containing corn, brewer’s rice, and wheat as a grain source in order to determine the effect of sorghum fractions on dry dog food sensory properties. The aroma compounds and flavor profiles of samples were similar with small differences, such as higher toasted aroma notes, and musty and dusty flavor in the mill-feed sample. A total of 37 compounds were tentatively identified and semi-quantified. Aldehydes were the major group present in the samples. The total volatile concentration was low, reflecting the mild aroma of the samples. Partial least squares regression was performed to identify correlations between sensory characteristics and detected aroma compounds. Possible relationships, such as hexanal and oxidized oil, and broth aromatics were identified. Volatile compounds were also associated with earthy, musty, and meaty aromas and flavor notes. This study showed that extruded dry dog foods manufactured with different red sorghum fractions had similar aroma, flavor, and volatile profiles.

## 1. Introduction

The pet food industry represents an important segment of the food industry that has experienced significant growth in recent years. The American Pet Products Association (APPA) reports $28 billion in sales for 2016, in the USA alone [1]. This industry is continuously experimenting with novel raw materials and, because pets are often treated as family members, there are trends from human foods that are being adopted into pet food products. 

Palatability of pet food products plays an important role for success on the market, but consumer acceptance of aroma and appearance of kibble needs to be understood as well [2]. Descriptive sensory analysis has been used to study sensory characteristics of dry and canned pet food products [2,3,4,5]. Pet foods can be complex in their characteristics as a wide variety of ingredients are utilized for different types of pet foods, such as dry, canned, or semi-moist products. With pet food, any given product must satisfy all of the nutritional requirements of the pet. The aromatic composition of products may reflect the ingredients used in the formulation. Ingredients utilized in dry dog food include meat ingredients, such as poultry, beef, and pork products, fresh, frozen, freeze-dried, deboned, or rendered meals. A variety of grains are also utilized as starch sources, mainly rice, wheat, soy, and corn. Other grains, such as barley and oats, are also utilized in smaller amounts. Sorghum represents one of the least utilized starch ingredients in pet foods [6]. Some studies found that in some sorghums may have some anti-nutritional properties, as condensed tannins may reduce feed efficiency due to interactions with starch [7] and protein digestibility [8]. Red sorghum is poorly utilized by the pet food industry because of the condensed tannin content, which is expected to impart bitterness to the products [9], and perhaps also because consumers often do not recognize this grain. 

The United States is one of the main producers of sorghum and Kansas is the leading producer in the USA. Due to its health benefits, such as its antioxidant properties, together with more environmentally sustainable agricultural practices [10], and potential marketing claims, sorghum could have potential for an increased use in human food and to become one of the carbohydrate sources utilized by the pet food industry. To understand if an increased utilization of sorghum is possible by this industry, it is essential to study aspects like the sensory characteristics of pet food manufactured with sorghum and its fractions. Currently, food manufacturers utilize whole sorghum and an investigation of the properties of the different sorghum fractions, such as flour and bran, may provide useful insights for the use of these fractions as new ingredients. Moreover, several studies have investigated the use of whole sorghum in extruded pet diets, but there are no published studies related to the use of the fractions for this scope.

Due to the complexity of pet food, the study of the aromatic composition can provide important information in order to understand the product [11]. Several studies exist that investigated the volatile aromatic composition of food, such as grains or meat ingredients, which can be part of pet food products as raw materials [12,13,14]. The analysis of the volatile compound composition of different types of grains, such as corn, rye, wheat, barley, and rice, has been conducted using extraction techniques such as solid-phase microextraction (SPME). This technique has also been utilized in the current study.

The objectives of this study were to: (1) detect the sensory properties and volatile compound composition of pet foods manufactured with sorghum fractions and compare those to the control, and (2) to understand the possible relationship between instrumental and descriptive sensory analysis data.

## 2. Results and Discussion

### 2.1. Volatile Components of Samples

A total of 37 aromatic compounds were tentatively identified among the four dry dog food diets. The concentration of each of the compounds, grouped by chemical family, and the total concentration of each chemical group have been reported in Table 1 and Table 2. The compounds were grouped as alcohols (six compounds), aldehydes (11 compounds), alkanes (11 compounds), alkenes (two compounds), carboxylic acids (two compounds), furans (one compound), hydroxy acid (one compound), ketones (four compounds), and terpenes (one compound). 

The total concentration of volatiles was similar among the four samples (Table 1 and Table 2) (1.78–2.09 µg/kg, average 1.89 µg/kg). All of the diets contained grains (about 60% of the total ingredients) and other common ingredients, such as chicken fat, beet pulp, and corn gluten meal. A similar total content of volatiles among samples was expected. In a study that identified volatile compounds of commercial extruded dry dog food products from the market, Koppel et al. [11] found an average of 22.07 µg/kg (10.60–41.34 µg/kg) for grain-added samples and 13.63 µg/kg (8.24–17.37 µg/kg) for grain-free samples. Those volatile concentrations were much higher than the ones found in the present study. The lower concentrations were likely caused by sample formulations that did not include any additional flavors or palatants.

Aldehydes were the most abundant group of volatiles detected in the samples, accounting for almost 50% of the total volatile compounds in each of the diets. Some differences were found among the samples for aldehyde content. The Whole Sorghum Diet (WSD) sample had the highest concentration (0.98 µg/kg) of aldehydes, followed by the Mill Feed (MF) sample (0.91 µg/kg), Control Diet (CD) sample (0.85 µg/kg), and Flour Diet (FD) (0.72 µg/kg). Aldehydes have been shown to play a major role in odor contribution even if present in low concentration, since aldehydes often have low thresholds, in the range of a few micrograms per liter of water [13]. Hexanal, the main product of oxidation of linoleic acid [15], was the most abundant compound among the aldehydes, with a concentration range varying from 0.33 µg/kg in the FD sample to 0.55 µg/kg in the MF sample. Odor notes associated with hexanal have been described as leaf-like [16], greenish [17], grass-like, and green tomato [18]. 

Other studies have identified volatile compounds in grains, such as oats. For example, Lampi et al. also found hexanal to be the abundant compound in the samples [23]. Lwande and Bentley identified volatile compounds present in sorghum seedlings and listed hexanal as the third most abundant compound preceded by (*Z*)-3-hexen-1-ol and (*Z*)-3-hexen-1-ol acetate [24]. Neither of these compounds were identified in the extruded samples in the current study, though.

Other aldehydes, such as benzaldehyde, isovaleraldehyde, 5-methylhexanal, furfural, and benzeneacetaldehyde, were present at lower concentrations. Benzaldehyde can be a thermal reaction product of hexanal and deca-2,4-dienal [25] and it has been identified in extruded cereals [26], cooked rice [27], and dry dog food products [11].

The group with the second highest concentration of volatiles was represented by alkanes. Alkanes can be formed during lipid oxidation from division of saturated alkoxy radicals. Aliphatic hydrocarbon compounds have high odor thresholds and do not usually play a major role in odor contribution [28]. Sample FD had the highest concentration of total alkanes (0.55 µg/kg) while sample MF showed the lowest (0.48 µg/kg). Specifically, sample FD had the highest content of 2,6-dimethylheptadecane (0.17 µg/kg).

Alcohols and ketones were also detected in smaller concentrations in all of the samples. Alcohols are formed by decomposition of the secondary hyperoxides of fatty acids [29]. These are generally associated with fruity, floral, and grassy notes in cereal. The two alcohol compounds with the highest concentration were 2-nonen-1-ol and 1-nonen-3-ol (0.07–0.09 µg/kg). The total alcohols concentration was lower in the MF sample compared to the other samples. 

Hydroxy and carboxylic acids, such as butyric acid, hexanoic acid, and 2,3,5-trimethoxymandelic acid, were also identified in all of the diets even if all of these were present at low concentrations (Table 2). In this study WSD sample had the highest concentration of carboxylic acids (0.06 µg/kg), while sample MF had the lowest (0.02 µg/kg). 

Hexanoic acid has been shown to be the major volatile compound in oat extrudates during extensive lipid oxidation, even more than hexanal, which is usually adopted as a lipid oxidation indicator [23].

During the extrusion of grain flours, it is possible to individuate two main reactions that lead to the formation of volatile compounds: Maillard reactions and lipid degradation. During the Maillard reaction, where several reactions between reducing sugars, amino acids, and their respective degradation, occurs, mostly desirable notes, such as toasted grain aroma notes are generated. From a compound generation standpoint, a common category of compounds generated during Maillard reactions are Strecker aldehydes, by decarboxylation and deamination of amino acids [14]. Off-flavors, associated with compounds such as hexanal and pentanal, are instead often the products of lipid degradation reactions. The volatile compounds produced by lipid degradation have been extensively described and are mostly represented by aliphatic aldehydes, alcohols, and ketones derived from fatty acids. Extrusion conditions can have an influence on both these type of reactions [14].

Koppel et al. [11] identified volatile compounds in extruded commercial dry dog food, manufactured with and without grain, and found that aldehydes were the major volatile group in the wide variety of samples analyzed. The most abundant compounds found in that study for grain based food were hexanal followed by benzaldehyde. Pyrazines, such as 2,5-dimethyl pyrazine, were found in some of the samples, both grain-based and grain-free. However, pyrazines were not detected in the samples analyzed in this current study. 

### 2.2. Aroma and Flavor Characteristics of Samples

Results from descriptive sensory analysis showed that significant differences (*p* < 0.05) among samples were found for toasted aroma, musty, and dusty flavor, with barnyard and brown aftertaste. Generally, the intensities for most attributes were in the low range of the scale (0–4.5). Similar findings have been found by Di Donfrancesco et al. [30] and Chanadang et al. [31] when analyzing sensory characteristics of dry dog food samples. 

The aroma profile was found to have little differences among samples with scores lower than 2.0 (on a scale from 0 to 15) except for barnyard aroma scores (2.3–2.73) (Table 3). The toasted aroma was found to be the only attribute that was significantly different among the samples. The MF sample had the highest value for the toasted aroma (score 1.70).

Low scores were also observed for flavor notes of the samples (Table 4). The only samples with a score > 2.0 were barnyard, cardboard, and bitter. Sensory attributes were similar for flavor, as well, with the only significant difference found in the mill-feed (MF) sample that showed a higher dusty flavor and a musty note. No differences were found for bitterness or astringency. 

The aftertaste notes showed some differences among diets (Table 5). The FD sample was lower than the other samples for barnyard aftertaste and the MF sample showed the highest brown aftertaste. In descriptive sensory analysis brown is often utilized to describe a sharp, caramel, almost-burnt aromatic (a part of the grain complex) [30].

Similar low scores in sensory attributes of dry dog food products have been noted by Di Donfrancesco et al. [30] who described dry dog food products as a highly-blended category with low flavor scores and often narrow ranges. However, this should also be interpreted by taking into account the specific methodology adopted, with 3 g of sample used for aroma analysis and the specific anchors on the scale used. Sensory attributes were similar for flavor, as well, with the only significant difference represented by the mill-feed (MF) sample that showed a higher dusty flavor and a musty note. 

The samples analyzed in the study were not perceived to be different for bitterness or astringency. Studies have indicated sorghum can cause bitter and astringent notes [9]. Results from this study showed that extrusion can limit these characteristics in the final product. This data is in agreement with findings from Cardoso et al. [32] who showed that proanthocyanidins, which can be responsible for higher bitterness and astringency, were reduced after extrusion. Further studies need to show how a reduced bitterness and astringency may contribute to the acceptance of extruded products by dogs. Dogs tolerate bitter tastes better than cats [33], but this does not mean that lower bitterness could add value to the product and increase palatability.

These results showed that dry dog foods that were manufactured with red sorghum, are quite mild in their aroma characteristics. This indicates potential for additional palatants to be added to the foods to make the diets more appetizing for the animals and also that there may not be a great need for flavor-masking applications.

### 2.3. Association of Sensory Attributes and Volatile Compounds

**Figure 1:**
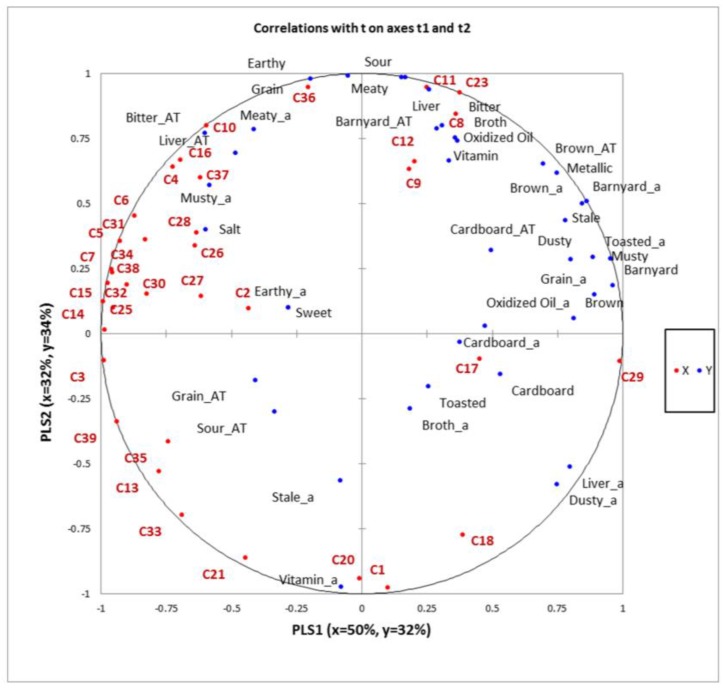
Partial least squares regression factors 1 (x = 50%, y = 32%) and 2 (x = 32%, y = 34%). X-matrix = chromatographic analysis and Y-matrix = descriptive sensory data. Red dots (C): volatile compounds from the chromatographic analysis; blue dots: sensory attributes from the descriptive sensory data (no suffix: flavor; a suffix: aroma; AT suffix: aftertaste).

The first two partial least squares factors explained 66% of the Y-matrix (descriptive data) variability and 82% of the X-matrix (instrumental data) variability (Figure 1). 

Several potential correlations were observed between sensory characteristics of the diets and volatile compounds detected. Compounds, such as *Z*-10-pentadecen-1-ol (C1), and 3,4-dimethyldecane (C20), were related to vitamin aroma. Further, octanal (C10) was correlated to different attributes in the samples (r ≥ 0.85), such as meaty aroma, grain, and earthy. From the literature [34] this compound has been associated with boiled meat, stewed, and rancid notes. For the same compound we also found associations with aromatic notes such as green, citrus, and flower [34]. Bryant et al. [18] described octanal to be also associated with citrus, fruity, floral, and fatty notes. As discussed by Chambers and Koppel [35], it is often difficult to associate volatile compounds with a specific sensory characteristic, as that may vary depending on the concentration of the compound, as well as the matrix the compound is in.

From the PLSR (Figure 1), benzeneacetaldehyde (C16), also known as phenyl acetaldehyde, was also associated with meaty aroma. However, from the literature this compound has previously been associated with green, clover, honey, and cocoa notes [20]. 

Hexanal (C8), has been described to be associated with green notes, and fat and tallow odors [34] and to be related to lipid oxidation [23]. Vazquez-Araujo et al. [36] also described this compound to be associated with fatty, fruity, and green notes. In the PLSR map (Figure 1) it is possible to observe that hexanal was actually related to oxidized oil and broth flavor in the samples.

A musty aroma was correlated to volatiles, such as 1-nonen-3-ol (C4) and (*E*,*E*)-3,5-octadien-2-one (C37). Different types of musty notes can be identified when performing descriptive analysis. These include musty dry, musty wet, and earthy/damp notes [37]. The type of musty note that was described in the descriptive analysis portion of this study was defined as “an aromatic that has a damp, earthy character similar to fresh mushrooms or raw potato”. The compound 1-nonen-3-ol has been actually described in literature as having earthy, mushroom, and green notes [34]. The compound (*E*,*E*)-3,5-octadien-2-one has also been associated in the literature with mushroom, woody, fresh, and sweet notes.

Cinnamaldehyde (C17) was close to toasted and cardboard aromatics contributing to the flavor of the samples. In the literature this volatile has been described as associated with notes such as cinnamon, spicy, and sweet aromatics [34]. Cinnamaldehyde, derived from the cinnamon tree, is the aldehyde responsible for flavor and aroma typical of cinnamon. Other than being used as food additive, cinnamaldehyde is also used as a fungicide in agriculture practices [38]. The Cinnamaldehyde concentration was slightly lower in the CD samples compared to the others. 

The PLSR map also showed that earthy aroma was associated with 4-methyl-1-pentanol (C2), an alcohol that showed the highest concentration, although still low, in the CD sample (0.03 µg/kg). In the literature this compound has been associated with nutty aroma notes [20].

### 2.4. Study Implications and Limitations

This study investigated volatile compounds that are associated with sensory attributes in different sorghum fractions when manufactured into dry dog food. The study gives the pet food industry important information on the potentially aromatic compounds that may result from using sorghum fractions in extruded dry dog foods. Some of the study limitations include the use of a SPME GC-MS, which tentatively identifies the volatile compounds in the sample headspace, but does not indicate whether the volatile has an aromatic or not. In addition most of the volatile compounds were detected in low concentrations and the quantification conducted was based on sample headspace volatile equilibrium. This may not reflect the actual aroma characteristics of these samples. Further, commercial dry dog food samples are usually coated with additional palatants or flavorings, while the samples in this study were not, in order to study the compounds that are derived from the raw ingredients alone. Future research may need to investigate whether these volatiles and sensory characteristics have an impact on the pet owner and pet acceptance of the products, as well as compare these results to aroma profiles of commercial products that utilize sorghum as an ingredient.

## 3. Materials and Methods 

### 3.1. Samples

#### 3.1.1. Milling process

Red sorghum used in the study was selected from locally-grown supplies in the Manhattan, Kansas, USA area during the 2013 and 2014 crop year. Sorghum was first cleaned of impurities, such as straw, weed seeds, soil particles, and dust. Then, most of the sorghum used in the study to manufacture samples was milled in April 2015 at the Hal Ross Flour Mill (HRFM; Kansas State University, Manhattan, KS, USA) in order to separate flour, bran (mill-feed), and germ. Sorghum moisture was conditioned with water to increase the moisture level to 16% from an initial 14% to promote the separation of the endosperm component from the germ and the hull. The milling process separated the different sorghum components according to particle size and consisted of grinding, sifting and purification steps. The grinding process consisted of five break passages that removed the endosperm from the bran portion and was collected in a bin. A purification step followed, where the bran was cleaned from any residual endosperm particles with the use of purifiers during the sifting process. The clean endosperm was then ground into flour. Germ was also collected and flattened into large flakes. To produce the whole sorghum meal used to manufacture the whole sorghum diet (WSD), the remaining portion was ground using a hammer mill (#16 standard sieve, 1.191 mm). After grinding, the sorghum was passed through a sifter sized with a 560 micron screen.

#### 3.1.2. Diet formulations

Four samples containing different sorghum fractions: whole sorghum (WSD), sorghum flour (FD), sorghum bran enriched mill-feed diet (MF), and the control diet (CD) made with corn, wheat, and brewer’s rice in a ratio of 1:1:1, were extruded in the Kansas State University facilities. The MF diet was composed of bran, shorts (finer bran), red dog (leftovers of the last flour cloth in the mill), and some coarse flour. Other than sorghum, rice, wheat, and corn, the diets also contained chicken by-product meal, beet pulp, corn gluten, calcium carbonate, potassium chloride, salt, dicalcium phosphate, choline chloride (60% dry), natural antioxidant (dry), trace minerals premix, and vitamin premix in order to have iso-nutritional diets (Table 6). 

Rendered chicken fat was provided from IDF (Springfield, MO, USA) and it was preserved with a commercial antioxidant added by the seller (BHA, propyl gallate, and citric acid). The additional ingredients were acquired from a local mill that supplies ingredients for pet food production (Fairview Mills L.P., Seneca, KS, USA). The diets were formulated in order to be iso-nutritional for carbohydrate, lipid, protein, and mineral content (Table 7). The methods used for the proximate analysis were based on AOAC International (Association of Official Analytical Chemists) and AOCS (American Oil Chemists’ Society) standard methods: moisture (AOAC 930.15); dry matter (calculation); protein (crude) (AOAC 990.03); fat (acid hydrolysis) (AOAC 954.02); fiber (crude) (AOCS Ba 6a-05); ash (AOAC 942.05); and micronutrients (AOAC 985.01–mod).

### 3.2. Extrusion

The mixing, grinding, and extrusion steps were conducted at the Bioprocessing and Industrial Value Added Program (BIVAP) facilities at Kansas State University, Manhattan, KS, USA. After being weighed with a digital scale ingredients were placed in a 227 kg paddle mixer. Micro-ingredients (<1% inclusion) were first mixed together and then added to the rest of the ingredients in the mixer. Ingredients were mixed for 5 min and then finely ground through a hammer mill with an 840 µm screen size to facilitate the next extrusion phase. 

**Figure 2:**
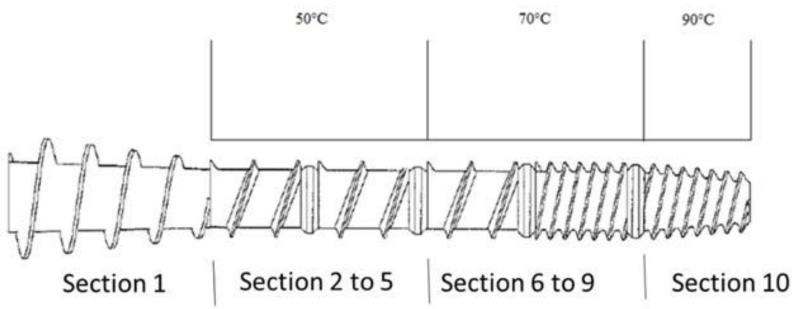
Extruder screw profile used to extrude the experimental diets control (CON), whole sorghum (WSD), flour (FLD) and mill-feed (MF).

For the extrusion of all the diets, a single screw extruder (Model X-20; Wenger Mfg, Sabetha, KS, USA) with a standard pet food screw profile was utilized. The adopted screw profile consisted of inlet screw, single flight full-pitch screw, small shear lock, single flight full-pitch screw, small shear lock, single flight screw, medium shear lock, double flight single pitch screw, large shear lock and double flight cut cone screw. The extruder screw profile and extrusion temperatures are shown in Figure 2. For the different diets, the measured temperature in the preconditioner was in a 97.1–98.5 °C range with the temperature for WSD (98.5 °C) being significantly higher (*p* < 0.05) than the temperature in the preconditioner recorded during MF extrusion (97.1 °C). After extrusion the kibbles were conveyed to a dual pass dryer/single pass cooler (Model 4800; Wenger Mfg, Sabetha, KS, USA) set at 99 °C in order to obtain a final moisture level lower than 10%. After the drying phase, the kibbles were transported in a coating tunnel where the addition of chicken fat occurred. The extruded diets were manually collected and placed in 9 kg poly-lined Kraft-paper bags.

### 3.3. Extraction Procedure of Volatile Aroma Constituents

Headspace-solid phase microextraction (HS-SPME) was the extraction method used to determine the aroma profile of the three dry dog food diets manufactured with red sorghum and the control diet. The samples were ground and then 0.5 g was weighed and placed in a 10 mL screw-cap vial (Supelco Analytical, Bellefonte, PA, USA) equipped with a polytetrafluoroethylene/silicone septum (Supelco Analytical, Bellefonte, PA, USA). Successively, 0.99 mL of distilled water was added to the ground sample in the vial. A similar approach was utilized by Koppel et al. [11]. The internal standard utilized was 0.01 mL of 1,3-dichlorobenzene dissolved in methanol (Sigma Aldrich, St. Louis, MO, USA) with a final concentration in the sample of 20 μg/kg. Vials were equilibrated in an autosampler (Pal system, model CombiPal, CTC Analytics, Zwingen, Switzerland) for 10 min at 40 °C and then agitated at 250 rpm. Following equilibration, a 50/30 μm divinylbenzene/carboxen/dimethyl-siloxilane fiber (Supelco Analytical, Bellefonte, PA, USA) was utilized. The fiber was exposed to the sample headspace for 30 min at 40 °C. After sampling, the analytes were desorbed from the SPME fiber coating to the injection port of gas chromatography (GC) at 270 °C for 3 min in splitless mode.

### 3.4. Chromatographic Analysis

Isolation, tentative identification, and semi-quantification of the volatile compounds were performed on a gas chromatograph (Varian GC CP3800; Varian Inc., Walnut Creek, CA, USA) coupled with a Varian mass spectrometer (MS) detector (Saturn 2000). The GC-MS system was equipped with a Stabilwax (Crossbond^®^ 5% Carbowax polyethylene glycol) column (Restek, U.S., Bellefonte, PA, USA; 30 m × 0.25 mm × 0.5 μm film thickness). The initial temperature of the column was 40 °C and it was held at this temperature for 4 min; the temperature was then increased by 5 °C per minute to 240 °C, and held at this temperature for 10 min. All of the samples were analyzed in triplicate. 

To identify most of the compounds two different methods were utilized: (1) mass spectra, and (2) Kovats indices (NIST/EPA/NIH Mass Spectral Library, Version 2.0, 2005, Gaithersburg, MD, USA), for pure chemicals. When only based on mass spectral data the identification was considered tentative. The retention times for a C7–C40 saturated alkane mix (Supelco Analytical, Bellefonte, PA, USA) was used to determine experimental Kovats indices for the volatile compounds detected. Additionally, pure chemicals were used in a mixture of chemicals to confirm the volatiles detected. These chemicals included 2-nonen-1-ol, 1-nonen-3-ol, isovaleraldehyde, hexanal, octanal, furfural, 5-methyl-2-furaldehyde, and 6-methyl-5-hepten-2-one (Sigma Aldrich, St. Louis, MO, USA).

### 3.5. Descriptive Analysis

Five highly-trained panelists from the Sensory Analysis Center, Kansas State University (Manhattan, KS, USA) analyzed the four sample diets for aroma and flavor characteristics. Each of the sensory panelists had more than 120 h of general descriptive sensory analysis panel training. The panel training included techniques and practices in attribute identification, terminology development, and intensity scoring. In addition, each panelist had experience with a variety of different food products including dried pet food, for both cats and dogs. 

An initial list of attributes from a sensory lexicon developed by Di Donfrancesco et al. [30] was utilized. The intensity of each attribute was evaluated on a 0–15 scale where 0 = none and 15 = very high. Panelists evaluated samples individually. Each sample diet was served in a ~100 mL plastic cup for flavor evaluation. For aroma evaluation, 3 g of each sample were placed in a medium glass snifter covered with a watch glass. Cups and snifters were coded with three-digit random numbers. The testing room was maintained at 21 ± 1 °C and 55 ± 5% relative humidity. The aroma and flavor attributes evaluated were barnyard, brothy, brown aromatics, cardboard, dusty, earthy, grain, liver, meaty, musty, oxidized oil, stale, toasted, vitamin. In addition, bitter, salt, sour, sweet, and metallic were also part of the pool of attributes. Aftertaste descriptors included barnyard, liver, brown, grain, cardboard, and bitter. The data from descriptive sensory analysis was used to associate volatile compounds to sensory characteristics through a partial least squares regression. All samples were analyzed in three replications.

### 3.6. Data Analysis

Analysis of variance (ANOVA) was performed (SAS version 9.4, The SAS Institute Inc., Cary, NC, USA) using PROC GLIMMIX and Fisher’s least significant difference (LSD) post-hoc means separation to determine statistical significance (*p* < 0.05) differences between the diets for sensory characteristics using samples as the fixed effect and panelist and replicate as the random effects. In addition, partial least square regression (PLSR) was performed to determine the correlation between instrumental data from the chromatographic analysis (X-matrix) and descriptive sensory data (Y-matrix). Other studies [11,39] also utilized the same approach when determining correlations between volatile compounds sensory characteristics of food. To perform PLSR, XLSTAT software was utilized (Addinsoft, New York, NY, USA).

## 4. Conclusions

Thirty-seven aromatic compounds were tentatively identified and semi-quantified in three extruded dry dog food samples manufactured with different red sorghum fractions and a sample manufactured with a combination of wheat, corn, and rice as grain sources. The total concentration of volatile compounds was similar across the different diets, as well as the concentration of the different volatile compounds groups. Aldehydes, especially hexanal, represented the main compounds in the samples. Partial least squares regression analysis showed some associations between sensory characteristics from the descriptive analysis and volatile compounds, such as hexanal with an oxidized oil and broth flavor, and octanal with a meaty aroma. The total concentration of volatiles detected in these samples was low compared with other studies that analyzed commercial dry dog food with a higher variety of ingredients and added flavors. Future studies using samples containing a higher variety in sensory characteristics will help to better understand relationships between sensory characteristics and volatile compounds in extruded dry dog food manufactured with different types of grains.

## Figures and Tables

**Table 1:** Content (µg/kg) of aroma compounds in the MF (Mill-feed), CD (Control Diet), FD (Flour Diet), and WSD (Whole Sorghum Diet). KI (Exp): experimental Kovats index, KI (Lit): Kovats index from the literature, SD: standard deviation.

Sample		KI Exp.	KI Lit.	MF	CD	FD	WSD
Code	Compound			Avg. ± SD	Avg. ± SD	Avg. ± SD	Avg. ± SD
Alcohols							
**C1**	Z-10-Pentadecen-1-ol	905	N/A	0.01 ± 0.00	0.01 ± 0.00	0.02 ± 0.00	0.01 ± 0.00
**C2**	4-Methyl-1-pentanol	N/A	1301 ^a^	0.01 ± 0.00	0.03 ± 0.01	0.01 ± 0.00	0.01 ± 0.00
**C3**	2-Nonen-1-ol	1218	1692 ^a^	0.07 ± 0.01	0.09 ± 0.02	0.09 ± 0.02	0.09 ± 0.02
**C4**	1-Nonen-3-ol	1237	1561 ^a^	0.07 ± 0.02	0.07 ± 0.01	0.07 ± 0.01	0.09 ± 0.00
**C5**	3-Furanmethanol	N/A	1649 ^a^	0.01 ± 0.00	0.02 ± 0.01	0.01 ± 0.00	0.02 ± 0.01
**C6**	Butanol, 4-(hexyloxy)-	1409	N/A	0.01 ± 0.00	0.01 ± 0.00	0.01 ± 0.00	0.02 ± 0.00
	Total Alcohols			0.18	0.24	0.21	0.25
Aldehydes							
**C7**	Isovaleraldehyde (3-methylbutanal)	N/A	920 ^a^	0.06 ± 0.02	0.08 ± 0.01	0.07 ± 0.02	0.08 ± 0.01
**C8**	Hexanal	N/A	1088 ^c^	0.55 ± 0.09	0.39 ± 0.09	0.33 ± 0.04	0.53 ± 0.12
**C9**	5-Methylhexanal	1325	1150 ^a^	0.06 ± 0.01	0.07 ± 0.03	0.04 ± 0.01	0.05 ± 0.03
**C10**	Octanal	1191	1291 ^c^ 1280 ^d^	0.04 ± 0.01	0.05 ± 0.01	0.04 ± 0.01	0.05 ± 0.00
**C11**	2- Heptenal (Z)-	1434	1336 ^b^ 1299 ^d^	0.02 ± 0.01	0.02 ± 0.00	0.01 ± 0.00	0.02 ± 0.00
**C12**	2-Octenal	1247	1437 ^a^	0.02 ± 0.00	0.01 ± 0.00	0.01 ± 0.00	0.02 ± 0.00
**C13**	Furfural	N/A	1432 ^c^	0.03 ± 0.01	0.06 ± 0.02	0.06 ± 0.01	0.05 ± 0.01
**C14**	Benzaldehyde	1538	1525 ^c^	0.07 ± 0.01	0.10 ± 0.01	0.09 ± 0.02	0.10 ± 0.01
**C15**	5-Methyl-2-furaldehyde	N/A	1591 ^a^	0.01 ± 0.01	0.03 ± 0.00	0.02 ± 0.00	0.03 ± 0.01
**C16**	Benzeneacetaldehyde	1306	1648 ^a^	0.02 ± 0.01	0.04 ± 0.00	0.02 ± 0.00	0.04 ± 0.00
**C17**	Cinnamaldehyde	1356	2044 ^a^	0.02 ± 0.00	0.01 ± 0.01	0.02 ± 0.01	0.02 ± 0.01
	Total Aldehydes			0.91	0.85	0.72	0.98

^a^: [19]; ^b^: [20]; ^c^: [21]; ^d^: [22].

**Table 2:** Content (µg/kg) of aroma compounds in the MF (Mill-feed), CD (Control Diet), FD (Flour Diet), and WSD (Whole Sorghum Diet). KI (Exp): experimental Kovats index, KI (Lit): Kovats index from the literature, SD: standard deviation.

Sample		KI Exp.	KI Lit.	MF	CD	FD	WSD
Code	Compound			Avg. ± SD	Avg. ± SD	Avg. ± SD	Avg. ± SD
Alkane							
**C18**	2,2,3-Trimethyldecane	650	N/A	0.02 ± 0.01	0.03 ± 0.00	0.03 ± 0.00	0.02 ± 0.00
**C20**	3,4-Dimethyldecane	876	N/A	0.03 ± 0.01	0.03 ± 0.00	0.04 ± 0.00	0.03 ± 0.01
**C21**	2,6-Dimethylheptadecane	N/A	N/A	0.11 ± 0.03	0.12 ± 0.02	0.17 ± 0.03	0.13 ± 0.04
**C23**	5-Ethyl-2,2,3-trimethylheptane	1013	N/A	0.15 ± 0.02	0.15 ± 0.03	0.14 ± 0.11	0.15 ± 0.06
**C25**	Hydroxylamine, *O*-decyl	1055	N/A	0.04 ± 0.00	0.04 ± 0.01	0.04 ± 0.02	0.05 ± 0.02
**C26**	2,3- Dimethyldecane	N/A	N/A	0.11 ± 0.04	0.11 ± 0.08	0.12 ± 0.02	0.13 ± 0.05
**C27**	Nonadecane	N/A	1900 ^a^	0.01 ± 0.00	0.01 ± 0.00	0.01 ± 0.00	0.01 ± 0.00
**C28**	4-Chloro octane	1283	N/A	0.01 ± 0.00	0.02 ± 0.01	0.01 ± 0.00	0.01 ± 0.00
	Total Alkane			0.48	0.50	0.55	0.52
Alkene							
**C29**	3-Dodecene, (*E*)	1202	1240 ^a^	0.03 ± 0.01	0.02 ± 0.00	0.02 ± 0.01	0.02 ± 0.01
**C30**	3,7-Dimethyl-1-octene	1503	963 ^a^	0.01 ± 0.00	0.01 ± 0.00	0.01 ± 0.00	0.01 ± 0.00
	Total Alkene			0.04	0.04	0.03	0.03
Carboxylic acid							
**C31**	Butyric acid	N/A	1628 ^c^ 1619 ^d^	0.01 ± 0.00	0.02 ± 0.01	0.02 ± 0.01	0.03 ± 0.01
**C32**	Hexanoic acid	N/A	1797 ^c^	0.01 ± 0.00	0.02 ± 0.00	0.02 ± 0.00	0.03 ± 0.00
	Total Carboxylic acid			0.02	0.03	0.04	0.06
Furans							
**C33**	2-Pentylfuran	1146	1239 ^a^	0.12 ± 0.04	0.14 ± 0.03	0.14 ± 0.04	0.13 ± 0.02
	*Hydroxy acid*						
**C34**	2,3,5-Trimethoxymandelic acid	N/A	N/A	0.02 ± 0.01	0.02 ± 0.00	0.02 ± 0.00	0.03 ± 0.00
Ketones							
**C35**	2,3-Octanedione	1207	1335 ^a^	0.01 ± 0.00	0.01 ± 0.00	0.01 ± 0.00	0.01 ± 0.00
**C36**	6-Methyl-5-hepten-2-one	1214	1341 ^a^	0.01 ± 0.00	0.01 ± 0.00	0.01 ± 0.00	0.01 ± 0.00
**C37**	(E,E)-3,5-Octadien-2-one	1274	1569 ^a^	0.02 ± 0.00	0.02 ± 0.00	0.02 ± 0.00	0.02 ± 0.00
**C38**	3,5-Octadien-2-one	1288	1569 ^a^ 1521 ^b^	0.01 ± 0.01	0.02 ± 0.00	0.02 ± 0.00	0.02 ± 0.00
	Total Ketones			0.05	0.06	0.06	0.08
Terpenes							
**C39**	Camphene	1157	1066 ^a^	0.01 ± (0.00)	0.01 ± 0.00	0.01 ± 0.00	0.01 ± 0.00
**Total**				1.81	1.89	1.78	2.09

^a^: [19]; ^b^: [20]; ^c^: [21]; ^d^: [22].

**Table 3:** Descriptive analysis for aroma of control (CD), whole sorghum (WSD), flour (FD) and sorghum mill feed (MF) containing diets with a trained human sensory panel.

Item	CD	WSD	FD	MF	*p*-Value
Barnyard	2.4 **	2.4	2.3	2.73	0.1561
Broth	1.5	1.17	1.33	1.33	0.2838
Brown	1.20	1.27	1.17	1.53	0.2208
Cardboard	1.93	1.73	1.80	1.87	0.5732
Dusty	1.07	1.07	1.27	1.27	0.4968
Earthy	0.10	0.07	0.07	0.07	0.9783
Grain	1.83	1.67	1.73	1.97	0.5456
Liver	0.10	0.10	0.37	0.40	0.2419
Meaty	0.93	0.90	0.70	0.80	0.6293
Musty	0.00	0.07	0.00	0.00	0.3997
Oxidized Oil	0.80	0.37	0.50	0.70	0.2208
Stale	0.43	0.20	0.37	0.27	0.6103
Toasted	1.43 ^a,b,^*	1.20 ^b^	1.23 ^b^	1.70 ^a^	0.0288
Vitamin	0.40	0.30	0.50	0.33	0.6474

*: Means with the same letter are not significantly different (*p* ≤ 0.05). Scores not sharing then same letter were significantly different (*p* ≤ 0.05). **: Scale 0 to 15 with 0.5 point increments.

**Table 4:** Descriptive analysis for flavor of control (CD), whole sorghum (WSD), flour (FD) and sorghum mill feed (MF) containing diets with a trained human sensory panel.

Item	CD	WSD	FD	MF	*p*-Value
Barnyard	2.90 **	2.83	2.87	3.03	0.7425
Bitter	3.70	3.93	3.67	3.93	0.1728
Broth	1.97	1.83	1.67	1.97	0.1480
Brown	1.83	2.00	2.03	2.23	0.1995
Cardboard	2.27	2.07	2.17	2.23	0.2790
Dusty	1.17 ^b,^*	1.40 ^b^	1.33 ^b^	1.70 ^a^	0.0063
Earthy	0.37	0.47	0.20	0.37	0.4397
Grain	2.30	2.33	2.17	2.30	0.7810
Liver	1.20	1.20	1.03	1.23	0.7934
Meaty	1.30	1.37	1.03	1.37	0.4366
Metallic	0.37	0.50	0.30	0.77	0.0946
Musty	0.00 ^b^	0.00 ^b^	0.00 ^b^	0.30 ^a^	0.0209
Oxidized Oil	1.27	1.17	1.03	1.27	0.7721
Salt	2.00	1.97	1.93	1.93	0.9238
Sour	1.70	1.73	1.60	1.73	0.7611
Stale	0.47	0.30	0.27	0.60	0.3853
Sweet	0.13	0.07	0.07	0.07	0.8134
Toasted	2.07	1.77	1.90	1.93	0.4406
Vitamin	1.10	0.93	0.80	1.07	0.3917

*: Means with the same letter are not significantly different (*p* ≤ 0.05). Scores not sharing then same letter were significantly different (*p* ≤ 0.05). **: Scale 0 to 15 with 0.5 point increments.

**Table 5:** Descriptive analysis for aftertaste of control (CD), whole sorghum (WSD), flour (FD) and sorghum mill feed (MF) containing diets with a trained human sensory panel.

Item	CD	WSD	FD	MF	*p*-Value
Barnyard	2.90 ^a,^*	2.63 ^a^	2.20 ^b^	2.87 ^a^	0.0023
Bitter	3.43 **	3.53	2.90	3.07	0.1766
Brown	1.10 ^b^	1.30 ^a,b^	1.03 ^b^	1.57 ^a^	0.0282
Cardboard	2.03	1.80	1.80	2.00	0.5706
Grain	1.53	1.77	1.73	1.60	0.7277
Liver	1.23	1.13	0.80	0.93	0.2504
Sour	1.17	1.00	1.07	1.00	0.6582

*: Means with the same letter are not significantly different (*p* ≤ 0.05). Scores not sharing then same letter were significantly different (*p* ≤ 0.05). **: Scale 0 to 15 with 0.5 point increments.

**Table 6:** Experimental diets composition. Control (CD), whole sorghum (WSD), sorghum flour (FD), and sorghum mill-feed (MF).

Ingredients, %	CD	WSD	FD	MF
Brewers’ rice	21.21	-	-	-
Corn	21.21	-	-	-
Wheat	21.21	-	-	-
Whole sorghum	-	64.69	-	-
Sorghum flour	-	-	62.31	-
Sorghum mill-feed	-	-	-	67.65
Chicken by-product meal	20.94	20.02	21.00	20.00
Chicken fat	5.34	5.52	5.52	3.29
Beet pulp	4.00	4.00	4.00	4.00
Corn gluten meal	3.00	3.00	3.00	3.00
Calcium carbonate	0.75	0.35	0.23	0.67
Potassium chloride	0.49	0.52	0.64	0.19
Salt	0.46	0.45	0.46	0.43
Dicalcium phosphate	0.87	0.95	1.19	0.24
Choline chloride	0.20	0.20	0.20	0.20
Vitamin premix	0.15	0.15	0.15	0.15
Trace mineral premix	0.10	0.10	0.10	0.10
Natural antioxidant (dry)	0.07	0.07	1.21	0.08
Natural antioxidant (liquid)	0.02	0.02	0.02	0.01

**Table 7:** Nutrient composition analysis of diets. Control diet (CD), whole sorghum diet (WSD), sorghum flour diet (FD), sorghum mill-feed diet (MF) (proximate analysis, Midwest Laboratory, Inc., Omaha, NE, USA).

Nutrient	CD	WSD	FD	MF
Moisture, %	7.20	6.46	6.44	9.56
Dry matter, %	94.67	94.31	95.08	94.29
Organic matter, %	91.55	93.10	93.10	92.42
Protein (crude), %	21.30	21.70	21.00	23.10
Fat (acid hydrolysis), %	12.80	10.60	10.20	9.80
Fiber (crude), %	0.57	1.69	1.07	3.13
Ash, %	8.45	6.90	6.90	7.58
Calcium, %	1.54	1.52	1.34	1.54
Phosphorus, %	0.85	0.94	0.86	0.88
Potassium, %	0.55	0.66	0.62	0.60
Magnesium, %	0.09	0.13	0.10	0.18
Sodium, %	0.28	0.28	0.27	0.24
Sulfur, %	0.28	0.28	0.26	0.28
Copper, ppm	15.50	16.60	15.00	16.50
Iron, ppm	168.00	177.00	156.00	181.00
Manganese, ppm	30.80	24.20	18.60	37.00
Zinc, ppm	132.00	141.00	131.00	144.00
